# Supplementary material for: The Triticum ispahanicum elongated glume locus P2 maps to chromosome 6A and is associated with the ectopic expression of SVP-A1
Source: Theor Appl Genet. 2022 May 18;135(7):2313–31. doi: 10.1007/s00122-022-04114-y (PMC9271103; doi:10.1007/s00122-022-04114-y)
Supplement: Supplementary file 2 — Supplementary file2 (PDF 1811 kb) [file 122_2022_4114_MOESM2_ESM.pdf]

Supplementary Figures:

The *T. ispahanicum* elongated glume locus *P2* maps to chromosome 6A and is associated with the ectopic expression of *SVP-A1*

Yi Chen[1], Yinqi Liu[1,2], Junli Zhang[3], Adam Torrance[1], Nobuyoshi Watanabe[4,5], Nikolai M. Adamski[1], Cristobal Uauy[1,*]

[1] John Innes Centre, Norwich Research Park, Norwich NR4 7UH, United Kingdom

[2] King's College London, Guy's Campus, London SE1 1UL, United Kingdom

[3] Department of Plant Sciences, University of California, Davis, California 95616, USA

[4] The Little Nursery, 1152 Ina, Toride, Ibaraki-PREF 302-0026, Japan

[5] College of Agriculture, Ibaraki University 3-21-1 Chuo, Ami, Inashiki, Ibaraki 300-0393, Japan

*Author for correspondence: cristobal.uauy@jic.ac.uk

Submission for Theoretical and Applied Genetics

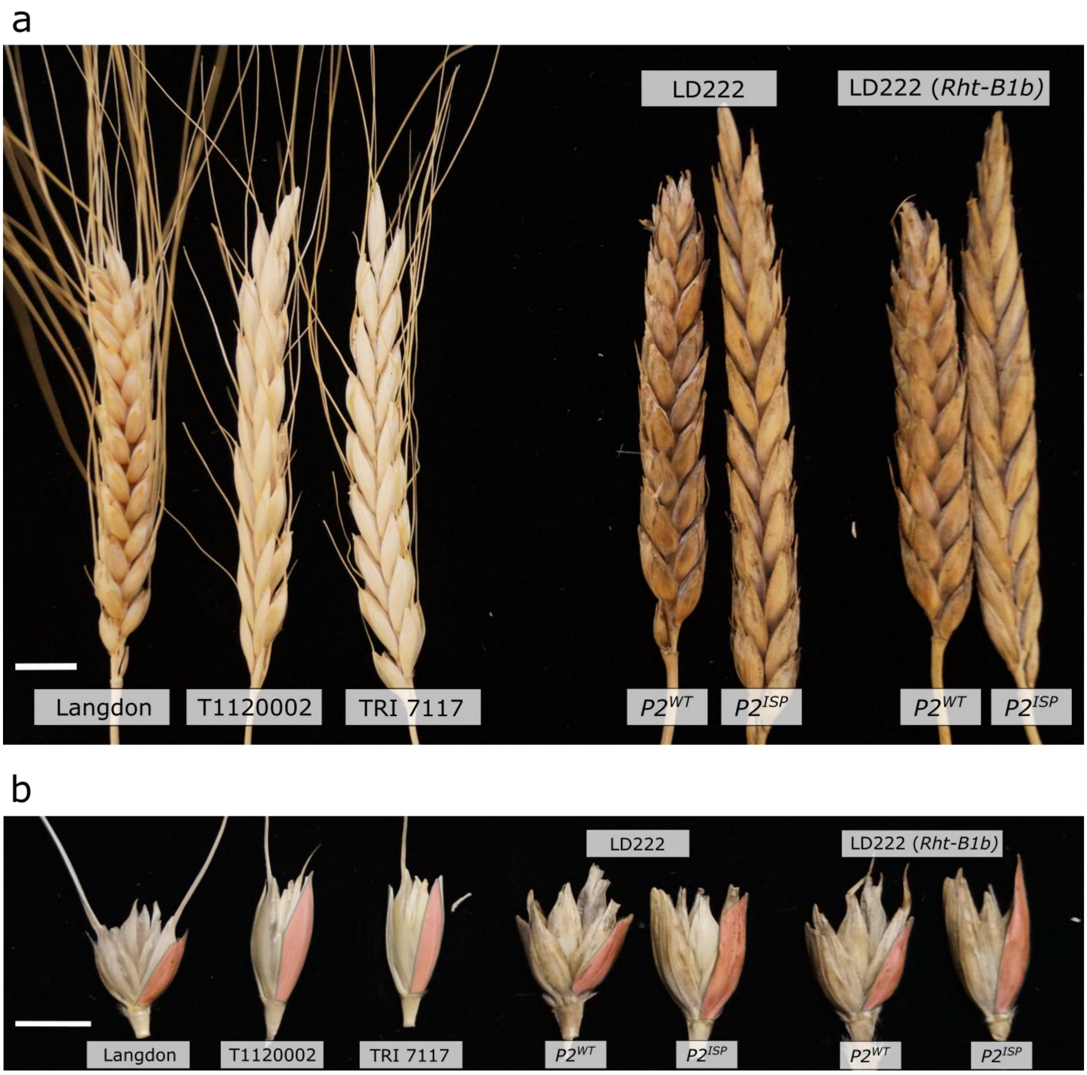

Fig S1 Spikes (a) and spikelet (b) of the parental tetraploid durum wheat cultivar Langdon, *T. ispahanicum* accessions T1120002 and TRI 7117, and the two sets of *P2* NILs used in the study. Scale bar = 1 cm. In b, glume tissues are outlined in red

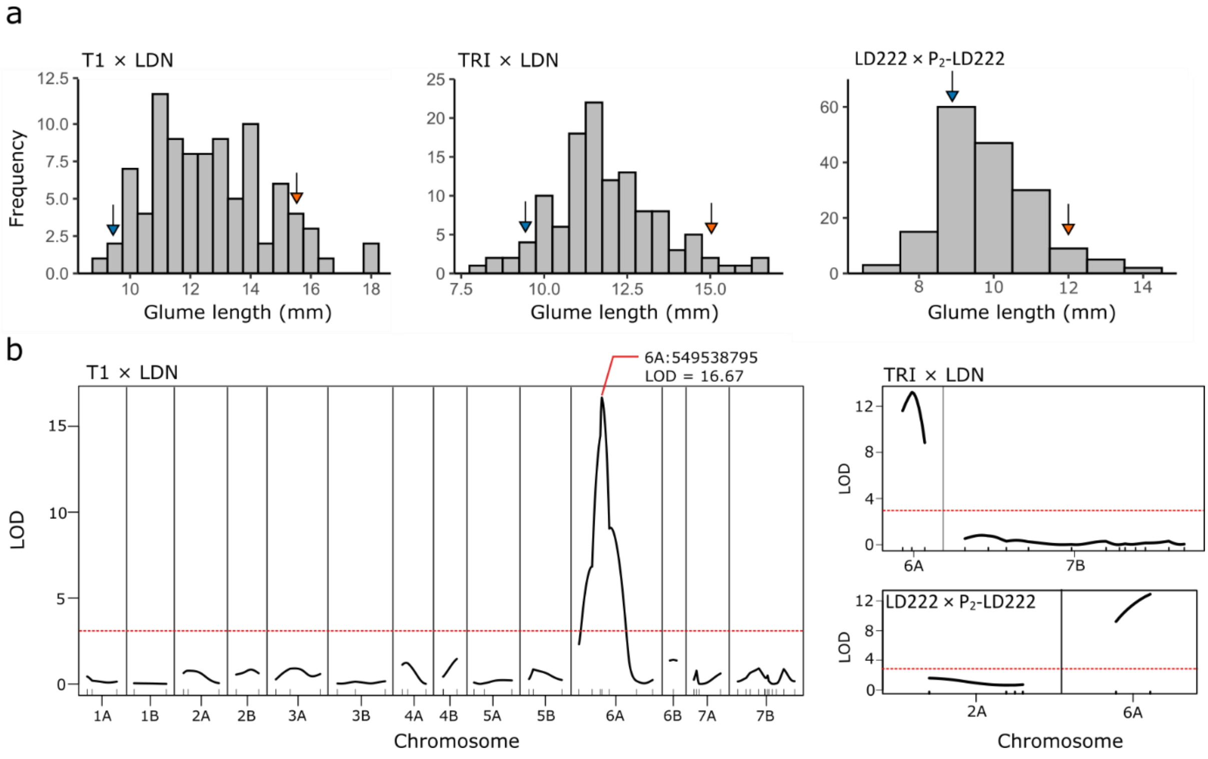

Fig S2 QTL analysis of glume length in three F_2 populations from the cross T1120002 (*T. ispahanicum*) × Langdon (T1 X LDN, *n* = 93 individuals), TRI 7117 × Langdon (TRI X LDN, *n* = 120 individuals), and LD222 × P2-LD222 (*n* = 172 individuals) **a**, Histograms of glume length variation in the three F_2 populations. Arrows indicate the glume size of *P2*^{ISP} parent (orange) and *P2*^{WT} parent (blue) grown in the same condition (n = 4 plants of each parental genotype). **b**, QTL plots of glume length for the three F_2 populations (significance threshold LOD > 3.0). The ticks represent individual markers reported in **Error! Reference source not found.**. Markers are ordered based on genetic position. For T1 X LDN, all chromosomes have at least three markers except chromosomes 1B (2 markers), 2B (2 markers), 4B (2 markers), and 6B (1 marker)

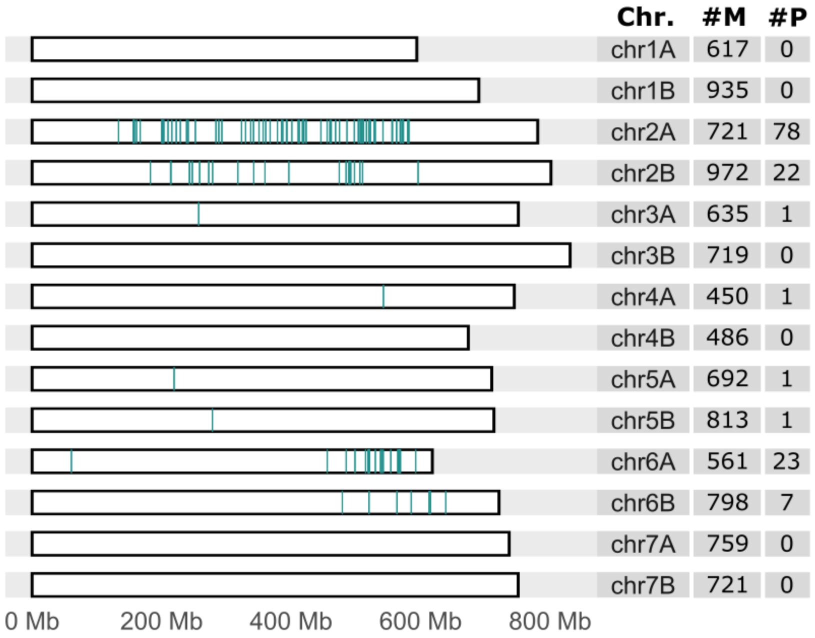

Fig S3 Polymorphic markers between the *P2* near-isogenic lines, LD222 and P2-LD222 using the 35K Axiom array. The distribution of polymorphic SNPs (teal vertical lines) between the *P2* NILs across the 14 chromosomes. #M represents the number of high-quality markers after filtering (see Materials and Methods; **Error! Reference source not found.**) on each chromosome and #P represents the number of polymorphic markers between the NILs.

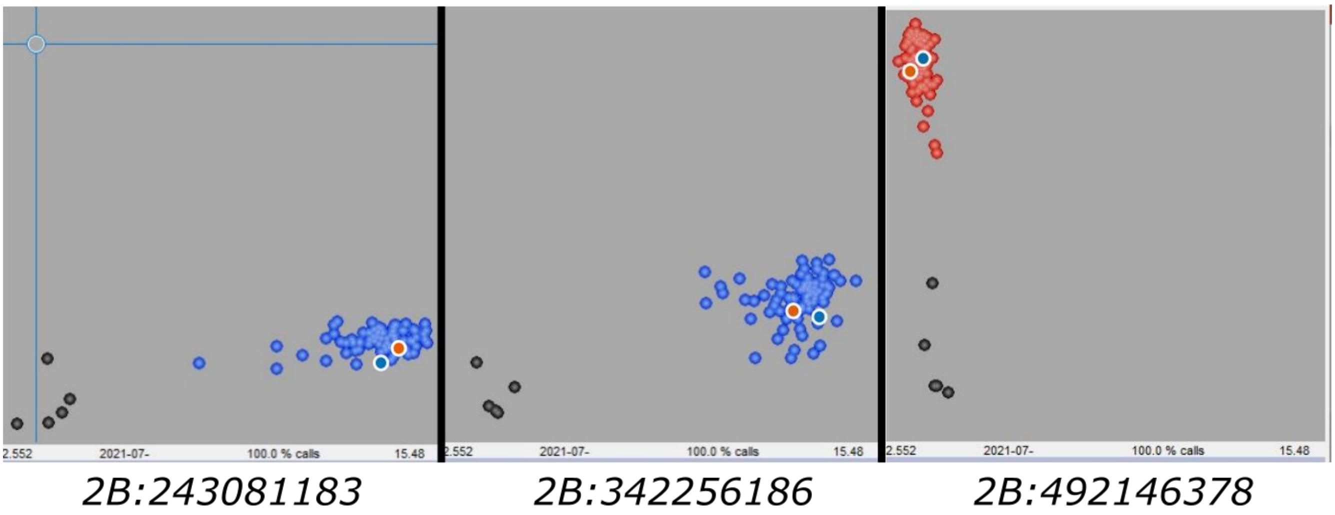

Fig S4 Cartesian cluster plot of the LD222 × P2-LD222 F_2 population genotyped with KASP markers from chromosome 2B. X-axis is the FAM value while the Y-axis is the HEX value. Black circles are no-template controls. The parents were included in the plate as controls and are highlighted by white circles filled in with blue (LD222) and orange (P2-LD222). The 2B markers identified as polymorphic via 35K Axiom array turned out to be monomorphic in the F_2 population

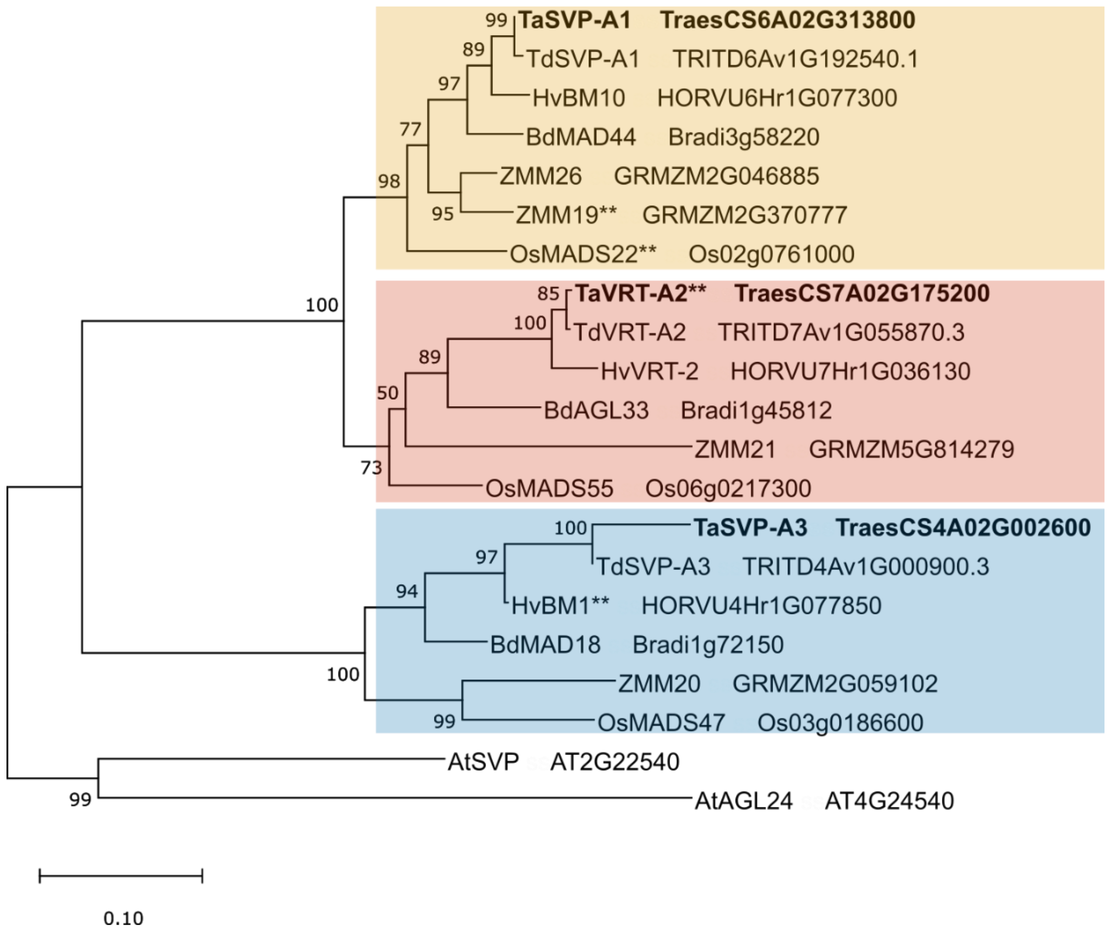

Fig S5 Phylogenetic tree of *SVP/StMADS11-like* proteins from *T. aestivum, T. durum*, *B. distachyon, H. vulgare*, *O. sativa*, and *Z. mays.* The three clades of *SVP/StMADS11-like* proteins in grasses are highlighted in different background colour where the proteins from *T. aestivum* are shown in bold. ** Genes that have been shown to influence glume or lemma length (Adamski et al. 2021; Sentoku et al. 2005; Trevaskis et al. 2007; Wingen et al. 2012)

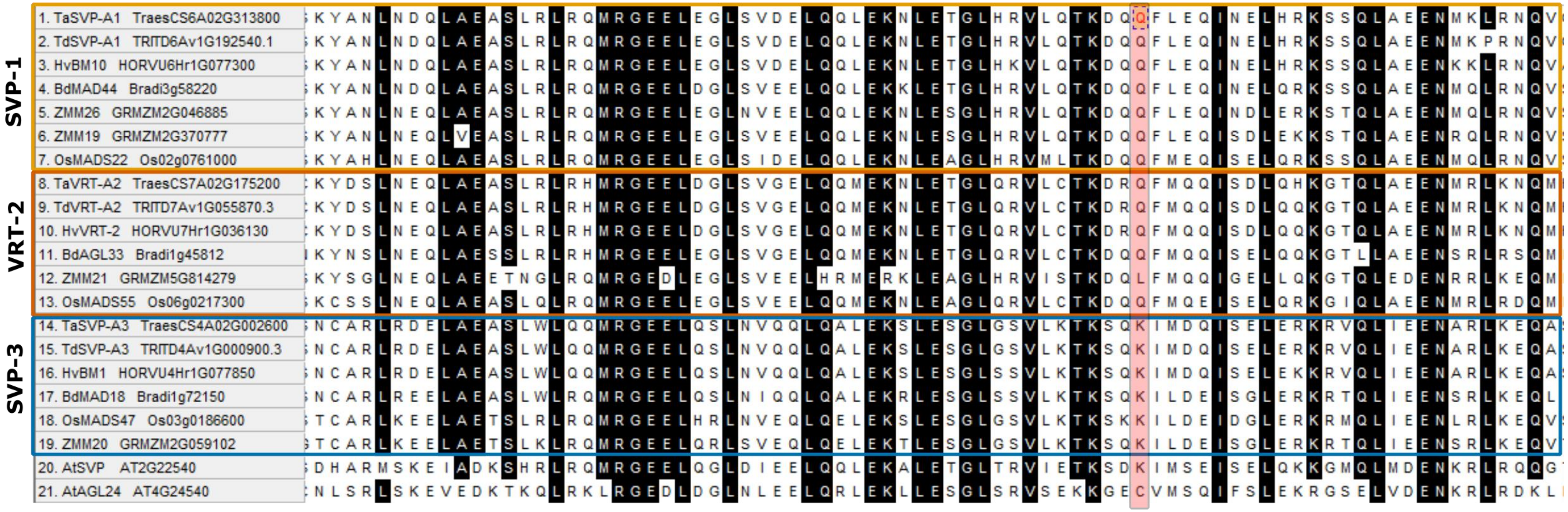

Fig S6 Alignment of the K-box domain (PF01486) of *SVP/StMADS11-like* proteins from *T. aestivum, T. durum*, *B. distachyon, H. vulgare*, *O. sativa,* and *Z. mays*. The alignment extends from amino acid 92 to 170 of SVP-A1. The three clades of *SVP/StMADS11-like* proteins (Fig S5) are indicated by coloured boxes; two *Arabidopsis* SVP proteins (*AtSVP* and *AtAGL24*) are also included in the alignment. The position of the Q144R missense mutation is highlighted in red. Amino acids that are conserved across 90% of the input sequences are highlight in black

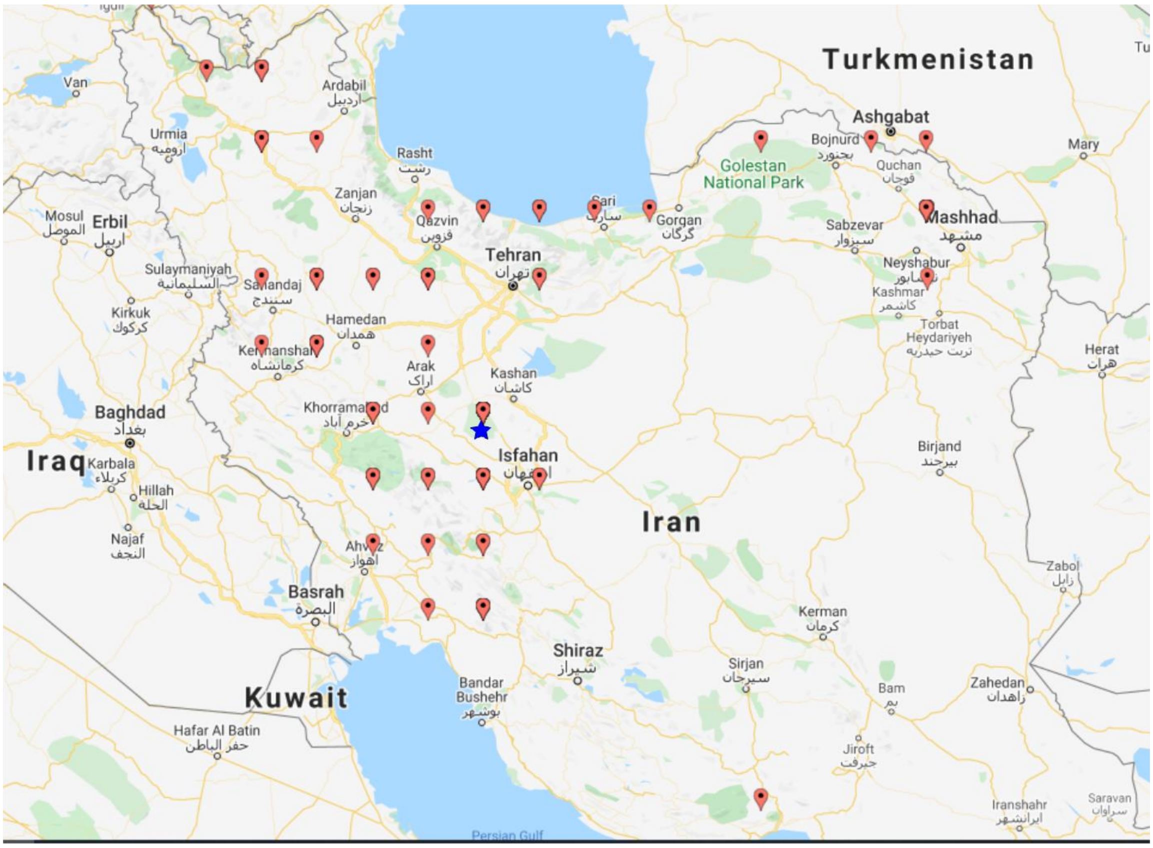

Fig S7. The recorded collection sites of wheat samples that were genotyped from the Kuckuck expedition (1952-1954). The blue star indicates where the *T. ispahanicum* accession samples were collected within the Isfahan province (Kuckuck 1956). Red flags indicate collection sites for 98 tetra- and hexaploid wheat accessions collected during the same expedition. Coordinates for these accessions are based on passport data from the IPK Genebank (**Error! Reference source not found.**)

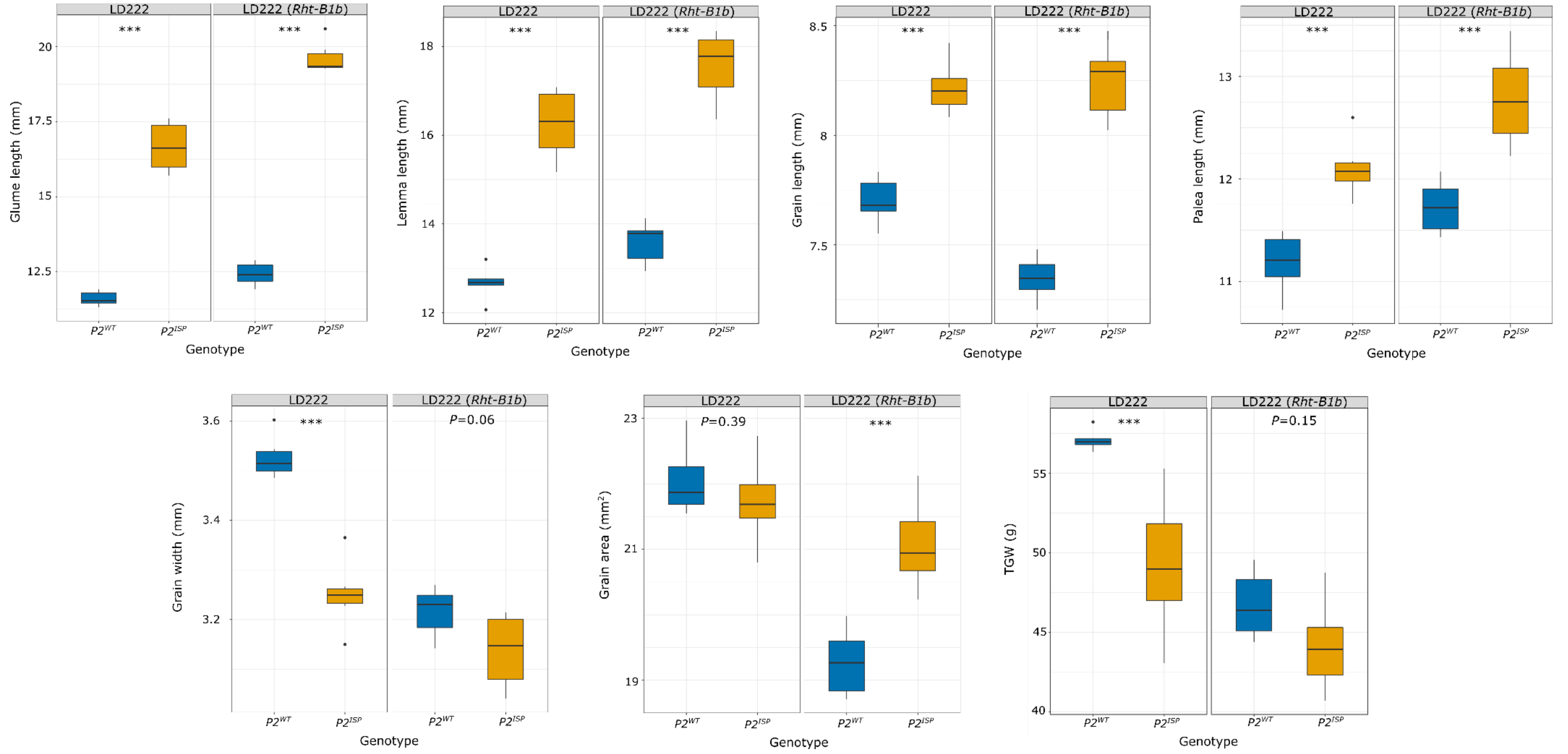

Fig S8 Phenotypic effect of *P2* on glume, floral organs, and grain morphology measured in LD222 and LD222(*Rht-B1b*) NILs grown in a randomized complete block design (*n* = 6 blocks) field trial. Measurements were performed on five representative primary spikes within each of the six biological replicates (blocks). Glume, lemma and palea length were based on the tissues from the first floret of the four central spikelets. LD222(*Rht-B1b*) NIL pair allow for the evaluation of *P2* effect under semi-dwarf background. Grain related traits were measured on all the grains from the five spikes using a MARVIN grain analyser. Thousand grain weight (TGW) was extrapolated based on grain weight of around ~200 grains. The box plots (blue for $P2^{WT}$; orange for $P2^{ISP}$) show the middle 50% of the data with the median represented by the horizontal line. Whiskers represent datapoints within 1.5 times the interquartile range while outliers are highlighted as individual dots. *P* values based on planned contrast of the phenotype performed between $P2^{WT}$ vs $P2^{ISP}$ within each background. **P* < 0.05; ****P* < 0.001

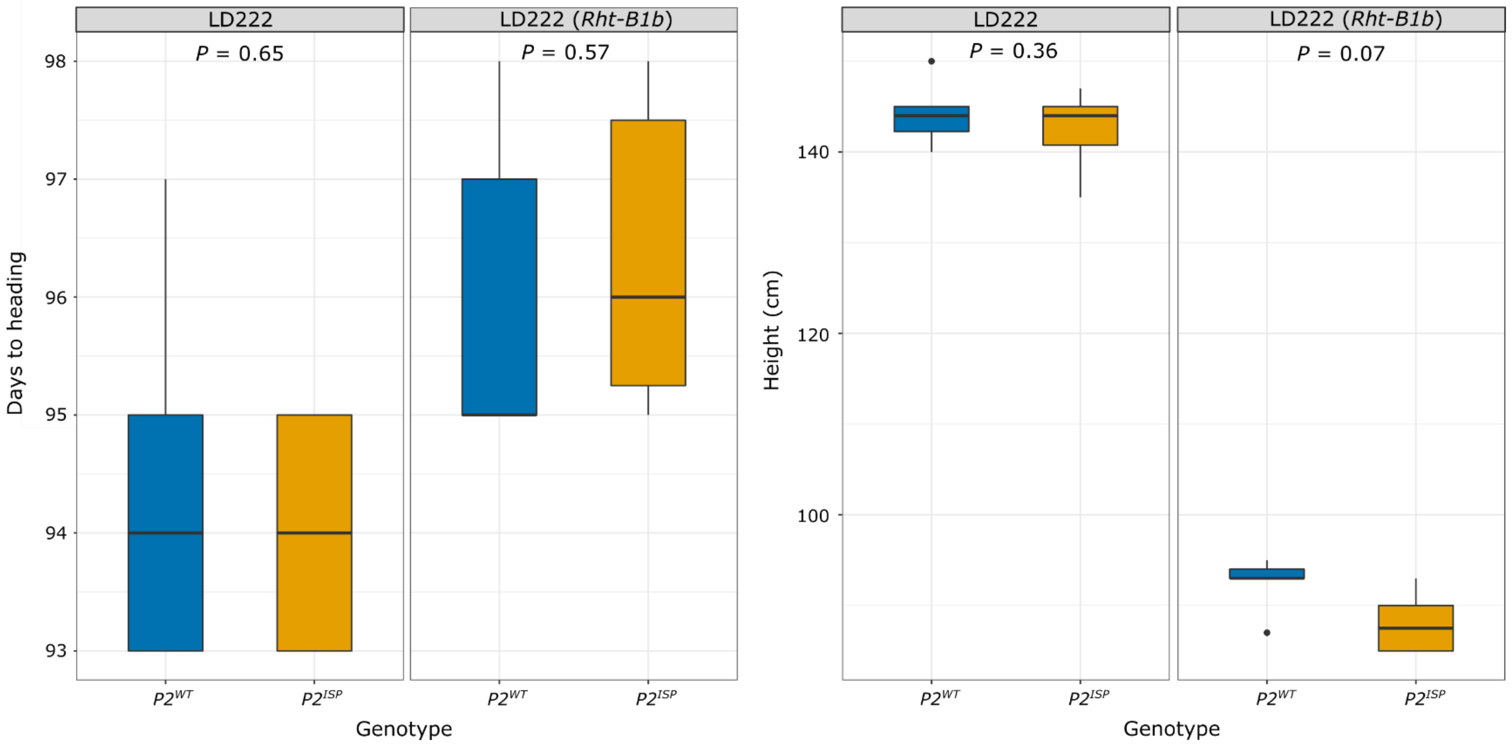

Fig S9 Effect of *P2* on phenology in field grown NILs in the LD222 and LD222(*Rht-B1b*) backgrounds. Measurements were performed on six biological replicates per genotype. LD222(*Rht-B1b*) NIL pair allow for the evaluation of *P2* effect under semi-dwarf background. The box plots (blue for $P2^{WT}$; orange for $P2^{ISP}$) show the middle 50% of the data with the median represented by the horizontal line. Whiskers represent datapoints within 1.5 times the interquartile range while outliers are highlighted as individual dots. *P* values based on planned contrast of the phenotype performed between $P2^{WT}$ vs $P2^{ISP}$ within each background

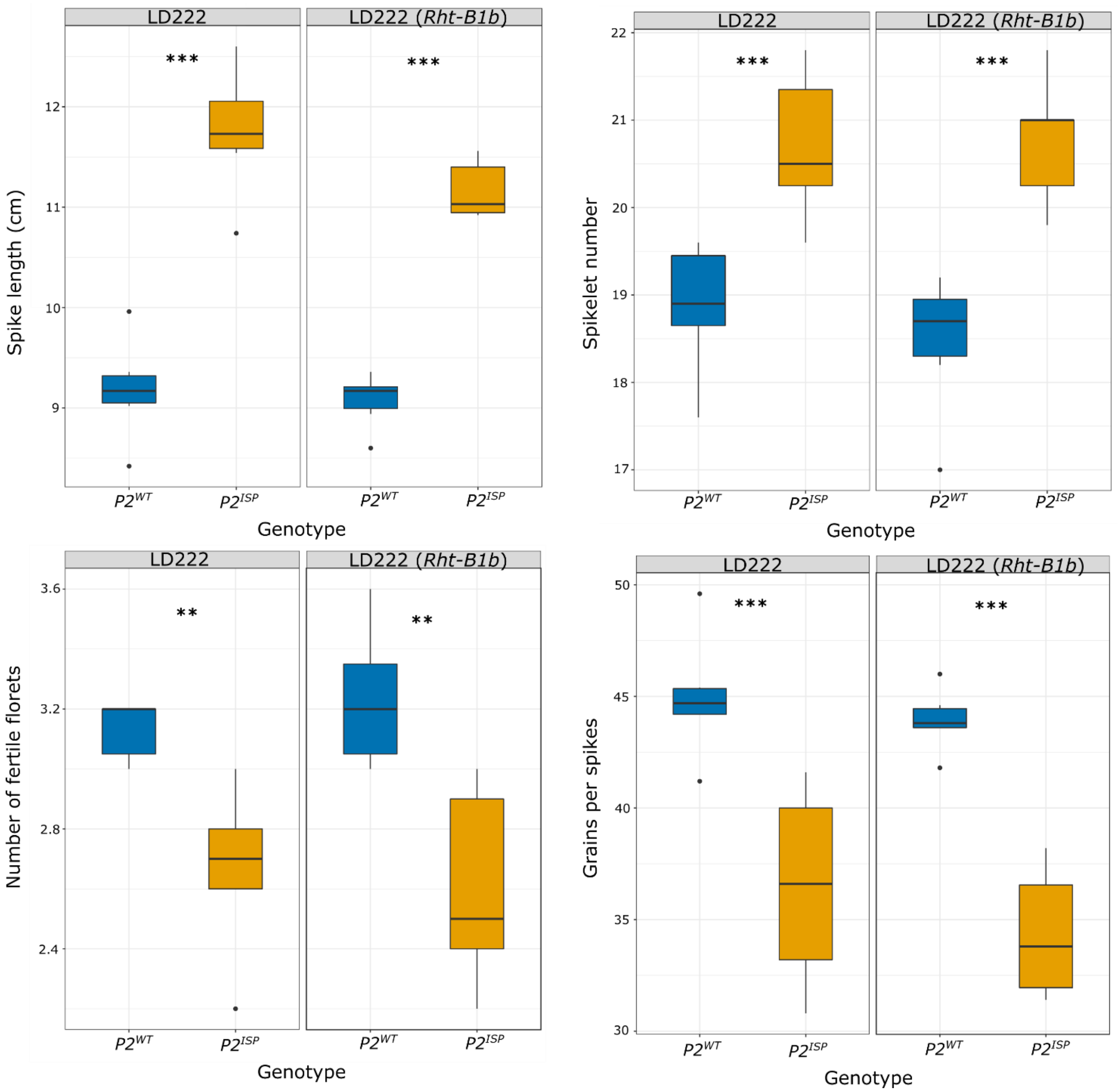

Fig S10 Effect of *P2* on spike morphology in field grown NILs in the LD222 and LD222(*Rht-B1b*) backgrounds. Measurements were performed on five representative primary spikes for each of the six biological replicates per genotype. The number of fertile florets is based on the number of grains per spikelet at two central spikelets. LD222(*Rht-B1b*) NIL pair allow for the evaluation of *P2* effect under semi-dwarf background. The box plots (blue for $P2^{WT}$; orange for $P2^{ISP}$) show the middle 50% of the data with the median represented by the horizontal line. Whiskers represent datapoints within 1.5 times the interquartile range while outliers are highlighted as individual dots. *P* values based on planned contrast of the phenotype performed between $P2^{WT}$ vs $P2^{ISP}$ within each background. $^{*}P < 0.05$; $^{**}P < 0.01$; $^{***}P < 0.001$

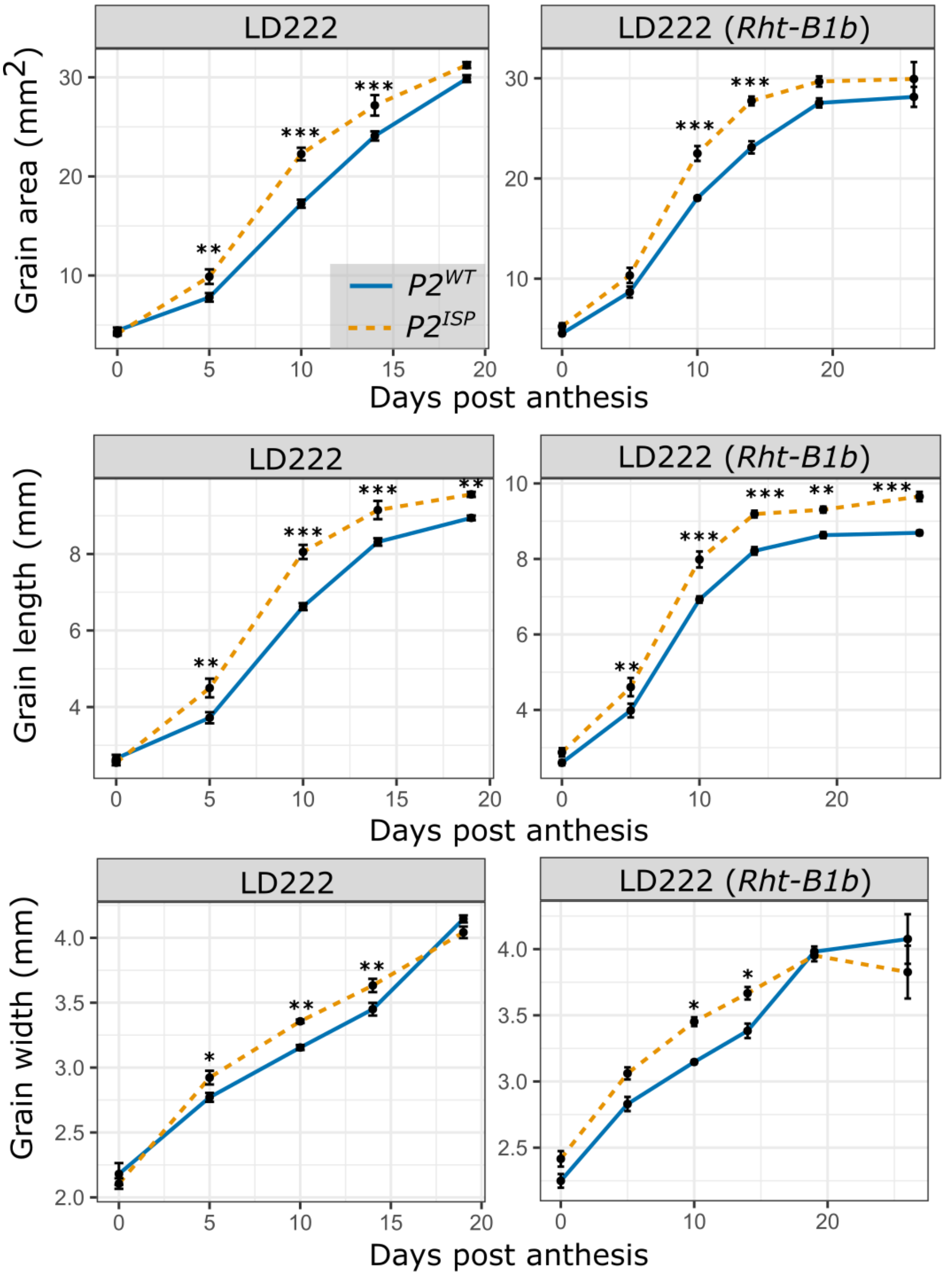

Fig S11 Grain development time course of field grown *P2* NILs in the LD222 and LD222(*Rht-B1b*) backgrounds. Measurements were performed on grains from five representative primary spikes for six biological replicates (blocks) per genotype (blue for $P2^{WT}$; orange for $P2^{ISP}$). The grains were collected from the first floret of four central spikelets for each spike. Data shows the average value of all biological replicates ± standard error of the mean. LD222(*Rht-B1b*) NIL pair allow for the evaluation of *P2* effect under semi-dwarf background. *P* values were based on planned contrast between $P2^{WT}$ and $P2^{ISP}$ within each pair of NILs at each timepoint. $^*P < 0.05$; $^{**}P < 0.01$; $^{***}P < 0.001$
